# Identification of leaf diseases in field crops based on improved ShuffleNetV2

**DOI:** 10.3389/fpls.2024.1342123

**Published:** 2024-03-11

**Authors:** Hanmi Zhou, Jiageng Chen, Xiaoli Niu, Zhiguang Dai, Long Qin, Linshuang Ma, Jichen Li, Yumin Su, Qi Wu

**Affiliations:** ^1^ College of Agricultural Engineering, Henan University of Science and Technology, Luoyang, China; ^2^ College of Water Resource, Shenyang Agricultural University, Shenyang, China

**Keywords:** complex background, crop leaf disease, ShuffleNetV2, EDCA module, residual structure

## Abstract

Rapid and accurate identification and timely protection of crop disease is of great importance for ensuring crop yields. Aiming at the problems of large model parameters of existing crop disease recognition methods and low recognition accuracy in the complex background of the field, we propose a lightweight crop leaf disease recognition model based on improved ShuffleNetV2. First, the repetition number and the number of output channels of the basic module of the ShuffleNetV2 model are redesigned to reduce the model parameters to make the model more lightweight while ensuring the accuracy of the model. Second, the residual structure is introduced in the basic feature extraction module to solve the gradient vanishing problem and enable the model to learn more complex feature representations. Then, parallel paths were added to the mechanism of the efficient channel attention (ECA) module, and the weights of different paths were adaptively updated by learnable parameters, and then the efficient dual channel attention (EDCA) module was proposed, which was embedded into the ShuffleNetV2 to improve the cross-channel interaction capability of the model. Finally, a multi-scale shallow feature extraction module and a multi-scale deep feature extraction module were introduced to improve the model’s ability to extract lesions at different scales. Based on the above improvements, a lightweight crop leaf disease recognition model REM-ShuffleNetV2 was proposed. Experiments results show that the accuracy and F1 score of the REM-ShuffleNetV2 model on the self-constructed field crop leaf disease dataset are 96.72% and 96.62%, which are 3.88% and 4.37% higher than that of the ShuffleNetV2 model; and the number of model parameters is 4.40M, which is 9.65% less than that of the original model. Compared with classic networks such as DenseNet121, EfficientNet, and MobileNetV3, the REM-ShuffleNetV2 model not only has higher recognition accuracy but also has fewer model parameters. The REM-ShuffleNetV2 model proposed in this study can achieve accurate identification of crop leaf disease in complex field backgrounds, and the model is small, which is convenient to deploy to the mobile end, and provides a reference for intelligent diagnosis of crop leaf disease.

## 1 Introduction

Various diseases in the process of crop growth will significantly reduce the yield and quality of agricultural products and seriously restrict agricultural production. To improve agricultural production efficiency, timely detection and early prevention of crop diseases are crucial (Hassan et al., 2021; Wang and Wang, 2021). At present, crop disease identification mainly relies on manual diagnosis, however the wide variety of crop diseases and the similarity of symptoms of some of them lead to a time-consuming and laborious diagnostic process (Barbedo, 2016). Image processing and machine vision can adapt to complex and changeable natural scenes and lay the foundation for accurate identification and diagnosis of crop disease (Zhang et al., 2014; Hossain et al., 2021; Ye et al., 2021). Therefore, computer vision and image processing strategies are utilized to design an intelligent recognition algorithm that can diagnose crop diseases quickly, inexpensively, and accurately, which is of great practical significance for the establishment of disease prediction mechanisms for timely prevention and control.

Since the 1980s, researchers have started to identify crop diseases using machine learning and image processing methods, proposing many traditional methods for image recognition of crop diseases (Camargo and Smith, 2009; Ma et al., 2017; Zhang et al., 2020). Tian et al. (2016) proposed a recognition method for eggplant brown streak disease based on spot characteristics, using the H component of the HSI color space to extract the feature parameters of the spot area and selecting the feature parameters to form a classification feature vector for classification by principal component analysis, which achieved better experimental results. Zhang and Zhang (2014) used region growing segmentation algorithm to segment disease spot images in diseased maize leaves and reorganized them into one-dimensional vectors, and used a nearest neighbor classifier to identify the disease categories with good recognition results. These traditional methods require manual design of features such as color, texture, and edge gradient of disease images for recognition. However, manually designed features require expensive resource conditions and specialized knowledge and are susceptible to subjectivity. In addition, the inability to efficiently segment leaves and corresponding disease images under complex background conditions has led to the inability of these methods to meet the needs of modern agriculture for accurate identification of crop disease.

In recent years, with the rapid development of deep learning techniques and the enhancement of computer processing power, crop leaf disease recognition methods based on convolutional neural networks (CNNs) have become a research focus of many researchers (Huang et al., 2021; Bao et al., 2022; Du et al., 2023; Praveen et al., 2023). Sun et al. (2021) embedded the coordinate attention mechanism in the MobileNetV2 model, and then performed fusion and extraction operations on feature maps of different sizes. The recognition accuracy of the improved model for a variety of crop leaf diseases was 92.20%. Rangarajan et al. (2018) used the strategy of fine-tuning and transfer learning for AlexNet and VGG16 to propose two fast converging models, which obtained 97.29% and 97.49% recognition rates on the tomato dataset. Gao et al. (2023) proposed an Apple Leaf Disease Recognition Model (BAM-Net) that uses an aggregated coordinate attention mechanism to enhance the network’s focus on disease features, introduces a multi-scale feature refinement module to improve the network’s ability to discriminate between similar disease features, which achieved an accuracy of 95.64% on the test set. Peng et al. (2022) introduced the SimAM module on the ShuffleNetv2 model to enhance the effective extraction of important features and used the activation function Hardswish to reduce the number of network model parameters, which resulted in a recognition accuracy of 84.9% on lychee pests and diseases. Bhagat et al. (2023) introduced local binary pattern for feature fusion based on the VGG-16 model and used random forest method for classification, which effectively improved the robustness of the model and achieved an accuracy of 99.75% on the sweet pepper leaf dataset. Agarwal et al. (2020) proposed a simplified convolutional neural network model that was tested on the tomato leaf dataset and the experimental results showed that the proposed model has better results than traditional machine learning methods. The above studies have proved the feasibility of CNNs in crop leaf disease recognition, but there are also problems such as a large number of network parameters, a large amount of calculation, and complex model, which make the model difficult to carry and move.

To solve the problem of mobile deployment of deep learning models, some researchers have proposed methods such as knowledge distillation and model pruning, aiming to improve the performance of network models and reduce the number of model parameters. Peng and Li (2023) proposed a plant leaf disease recognition model RLDNet based on improved MobileNetV2. The model used the reparameterized inverted residual module to improve the inference speed. The DepthShrinker pruning method is used to reduce the space occupation. The recognition accuracy of the RLDNet model on the PlantVillage dataset under simple background is 99.53%, and the number of parameters is 0.65 M. Liu et al. (2023) used the ResNet model as the baseline model, introduced a multi-teacher joint distillation strategy to train the model, and utilized model pruning to reduce the number of model parameters. After pruning the model by 90%, the model achieved up to 97.78% accuracy on the PlantVillage dataset, while after pruning the model by 70%, the model achieved up to 91.94% accuracy on the Apple Leaf Disease dataset in a complex context. Wen et al. (2023) used ShuffleNetV2 as the base network, introduced the efficient channel attention mechanism with the silu activation function for structural improvement, and also combined the knowledge distillation technique to train the model. The improved model achieved 95.21% accuracy in recognizing 11 diseases of two crops in a complex environment. However, although the above methods make the crop leaf disease recognition model lightweight, the effect of disease recognition in real scenes needs to be improved.

Based on the above problems, this study constructed a variety of crop disease datasets contained in the field context, and then used ShuffleNetV2-1.0 network as the baseline model, fine-tuned the model parameters, and introduced the efficient dual channel attention (EDCA) module, the multi-scale feature fusion module, and residual structure connection strategy. We propose a field crop leaf disease recognition model-REM-ShuffleNetV2 based on improved ShuffleNetV2. This model can effectively extract the subtle features of crop leaf diseases and improve the accuracy of crop disease classification in the field. Meanwhile, the model has the advantages of small size and few parameters, which can provide a reference for subsequent related research. The main innovations of this paper are as follows:

1. A lightweight CNN model REM-ShuffleNetV2 is proposed for the automatic identification of leaf diseases in field crops on mobile devices.
2. The number of repetitions and output channels of the basic module of the ShuffleNetV2 model are fine-tuned to reduce the model parameters and make the model lightweight.
3. The EDCA module is embedded in the basic feature extraction module, which enhances the model’s ability to extract effective feature information in crop disease, and introduces residual structure to alleviate the problem of information loss and gradient loss in the model.
4. A multi-scale shallow feature extraction module and a multi-scale deep feature extraction module are designed to enable the model to capture feature information at different scales, thus improving the model’s perceptual and expressive capabilities.

## 2 Datasets

### 2.1 Data acquisition

**Figure 1:**
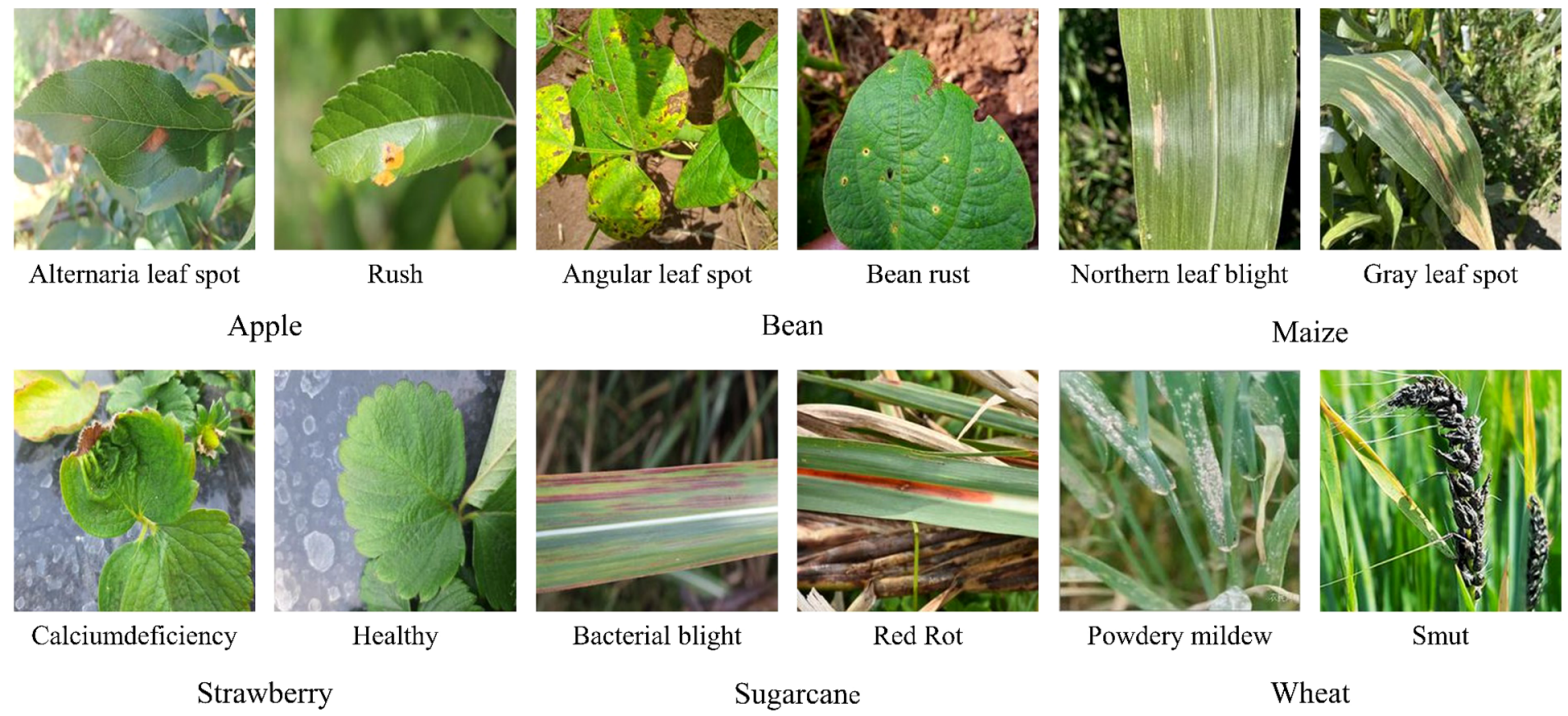
Diseased images of crops in a field background.

In this study, the dataset used contains 17 categories of diseased leaf images of six crops (apple, soybean, maize, strawberry, sugarcane, and wheat) and healthy leaf images of five crops (apple, soybean, maize, strawberry, and sugarcane), totaling 22 categories and 8,408 sample images from the field collection, the official website of Kaggle(https://www.kaggle.com/), and the website of Baidu Fly Paddle(https://aistudio.baidu.com/), and the sample images were all taken in a field background Photographed (Muhab and Ercan, 2022). Disease types include apple alternaria leaf spot, bean angular leaf spot, maize northern leaf blight, strawberry calciumdeficieny, sugarcane red rot, wheat powdery mildew, etc. Some sample images are shown in **Figure 1**.

### 2.2 Data set segmentation and preprocessing

The original dataset is randomly divided into a training set and a test set in a ratio of 8:2 (Liu and Cui, 2023), where the training set has 6732 images and the test set has 1676 images. To increase the diversity of crop disease datasets, and enhance the generalization ability and robustness of the model, this study performs data enhancement on the training set (Shorten and Khoshgoftaar, 2019). Data enhancement follows the principle of increasing the number of samples while keeping the sample features unchanged to better reflect the real background. In this study, two image enhancement techniques were used: 1) Brightness enhancement and attenuation: used to simulate different lighting conditions in real field background; 2) Rotation and flip: used to simulate the shooting of the recognition device at different angles. Finally, a sufficient and balanced training set with 22217 images is obtained by the augmentation technique. Detailed sample information is shown in **Table 1**.

**Table 1:** Detailed sample information on the dataset.

Crops	Types of diseases	Number	Before	After	Test set
Apple	Alternaria leaf spot	A1	219	1095	54
	Grey spot	A2	131	1048	32
	Health	A3	516	1032	129
	Mosaic	A4	137	1096	34
	Powdery mildew	A5	549	1098	137
	Rust	A6	447	894	111
	Scab	A7	477	954	119
Bean	Angular leaf spot	B1	264	1056	66
	Bean rust	B2	264	1056	66
	Healthy	B3	264	1056	66
Maize	Gray Leaf Spot	M1	398	1194	99
	Health	M2	265	1060	66
	Northern leaf blight	M3	419	1257	104
	Northern leaf spot	M4	441	882	110
Strawberry	Calciumdeficieny	S1	378	1134	94
	Healthy	S2	369	1107	92
Sugarcane	Bacterial blight	SU1	80	640	20
	Healthy	SU2	80	640	20
	Red rot	SU3	80	640	20
Wheat	Powdery mildew	W1	208	1040	51
	Smut	W2	330	990	82
	Rust	W3	416	1248	104
Total	–	–	6732	22217	1676

“Before” represents the original training set; “After” represents the augmented training set.

### 2.3 The process of disease identification

**Figure 2:**
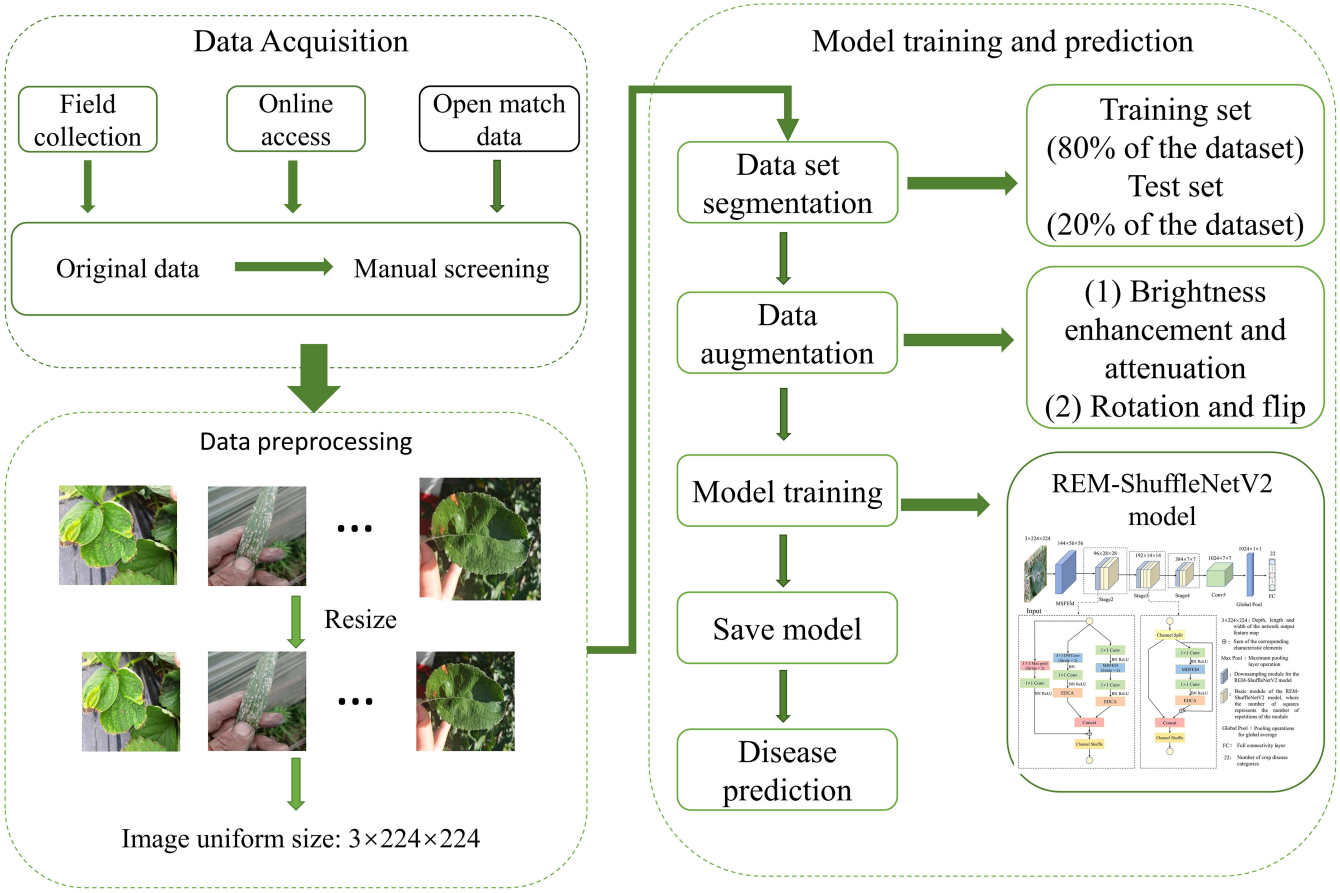
The overall process of disease identification.

The overall process of crop leaf disease identification is shown in **Figure 2**. Firstly, the disease image data were collected through multiple channels and the useless images were manually removed. Secondly, the constructed dataset is preprocessed and divided into training and testing sets in 8:2 ratio, and the original dataset is expanded by data enhancement to increase the diversity to improve the generalization ability of the trained model. Finally, the data-enhanced dataset is used to train the REM-ShuffleNetV2 model and the model weights with the best performance during training are saved. Based on the above trained REM-ShuffleNetV2 model, the images in the test set are used to get the prediction categories of the test samples for crop disease recognition on leaves. If more disease image data is subsequently collected, all can follow this process to retrain the model to improve the performance.

## 3 Crop leaf disease recognition model

### 3.1 ShuffleNetv2 model

**Figure 3:**
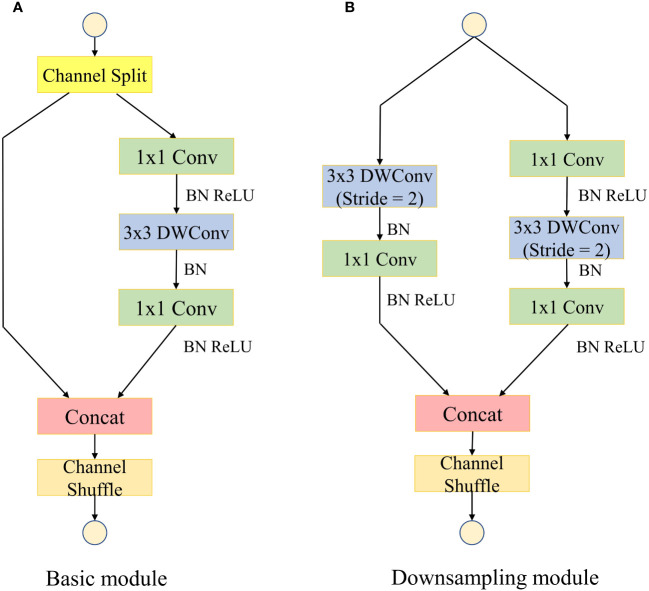
Basic feature extraction module for the ShuffleNetV2 model. “Conv” represents standard convolution; “BN” represents batch normalization; “ReLU” represents activation function; “Concat” represents channel splicing. **(A)** Basic module. **(B)** Downsampling module.

With the rapid development of convolutional neural networks in the field of computer vision, although the traditional convolutional neural networks have good accuracy, their large number of model parameters is difficult to adapt to today’s mobile devices with limited computational resources (Liu et al., 2017). ShuffleNetV2 is an extremely efficient lightweight convolutional neural network for mobile devices proposed by Ma et al. (2018). The network introduces the concept of group convolution which divides the input and output channels into multiple groups and performs convolution operations within each group. This design enables the network to parallelize processing efficiently and significantly reduce the computational cost. By rearranging the feature channels, information from different channels can be mixed and exchanged, leading to better representation learning and reducing the overall complexity of the network. The basic feature extraction module of ShuffleNetV2 is shown in **Figure 3**.

### 3.2 EDCA module

**Figure 4:**
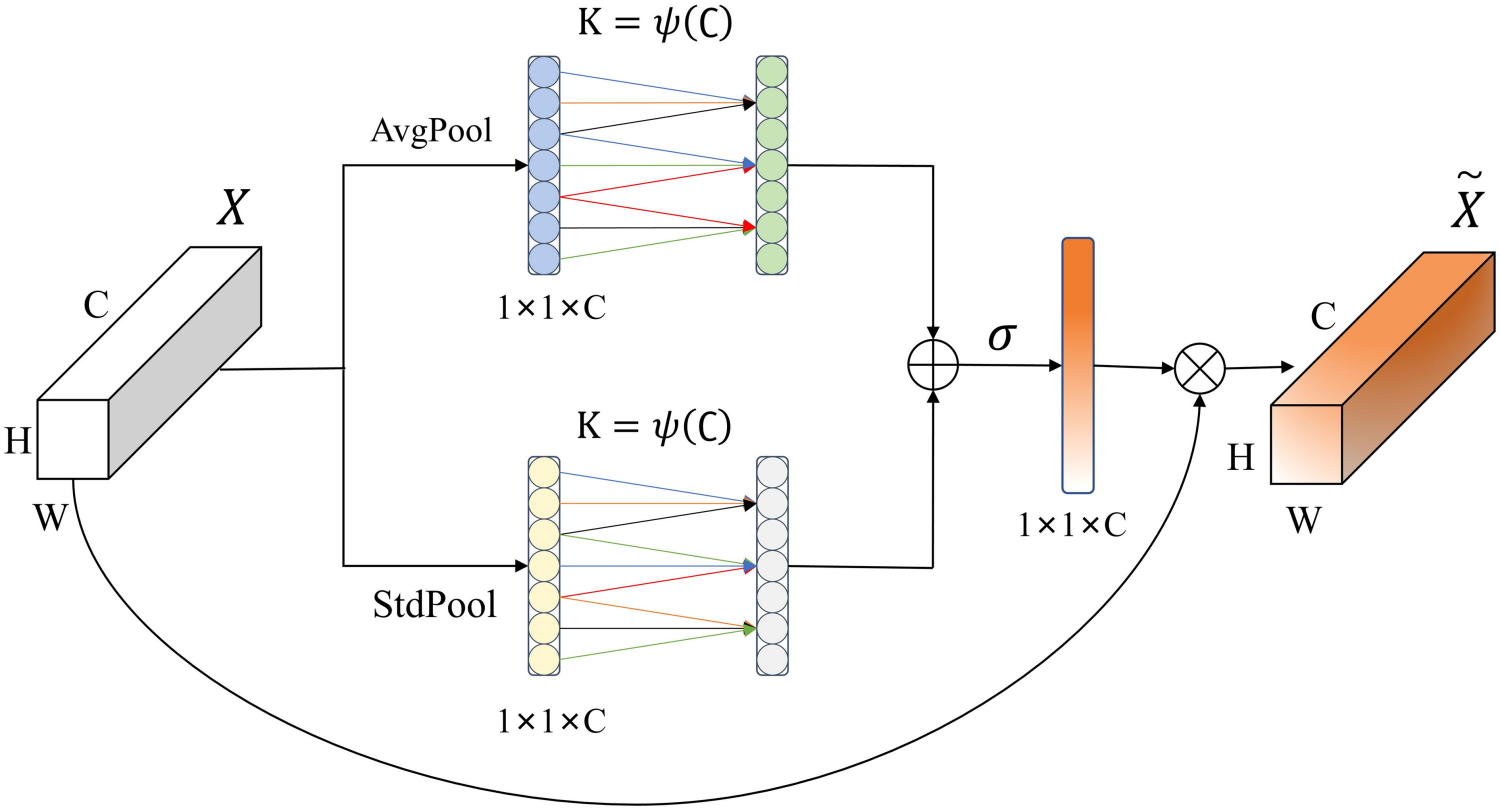
EDCA Module.

The crop disease samples in the dataset constructed in this study were taken in a field environment with complex background information. The attention mechanism adjusts the weight of the input feature map to suppress redundant background information and enhance the feature representation of the foreground disease in the image, thereby improving the recognition performance of the model (Huang et al., 2023). SE (Squeeze and Excitation) module uses global average pooling to aggregate global information, and then captures nonlinear cross-channel interactions by compressing channels for dimensionality reduction, but this approach is not conducive to learning inter-channel dependencies (Glorot et al., 2011). The ECA (Efficient Channel Attention) module uses one-dimensional convolution to realize cross-channel interactions and learns inter-channel dependencies while keeping the channel dimensions unchanged, and the model requires fewer parameters and less computation to introduce the ECA module compared to the SE module (Wang et al., 2020). To further optimize the global information extraction capability of the ECA module, inspired by the SRM (Style-based Recalibration Module) module (Lee et al., 2019), this study proposes an EDCA (Efficient Dual Channel Attention) module, and its structure is shown in **Figure 4**.

Suppose X is the input feature, and the size of the feature map is H×W×C, where H represents the height of the feature map, W represents the width of the feature map, and C represents the number of channels of the feature map. The EDCA module processes the input using average pooling (AvgPool) and standard deviation pooling (StdPool) to compress it into 1×1×C feature maps, respectively, and generates weights for each channel by one-dimensional convolution of size K. The average value and standard deviation are calculated as shown in (Equations 1, 2):


(1) $$A_{c}=\frac{1}{HW}\sum_{h=1}^{H}\sum_{w=1}^{W}X_{c}\left(\text{h},\text{w}\right)$$

(2) $$S_{c}=\sqrt{\frac{1}{HW}\sum_{h=1}^{H}\sum_{w=1}^{W}{\left(X_{c}\left(\text{h},\text{w}\right)-A_{c}\right)}^{2}}$$

In (Equations 1, 2), Ac and Sc represent the average value and standard deviation of each element in the channel.

The convolution kernel size K can be adaptively determined by nonlinear mapping of the channel dimensions, and the adaptation function is defined as shown in (Equation 3):


(3) $$\text{K}=\text{ψ}\left(\text{C}\right)={\left|\frac{log_{2}\left(\text{C}\right)}{\text{γ}}+\frac{b}{\gamma }\right|}_{\text{odd}}$$

In (Equation 3), C represents the input feature channel dimensions, |x|odd represents the closest singularity to x, γ and b are used to change the ratio between the number of channels C and the convolution kernel size, and are taken to be γ = 2 and b = 1 according to empirical values taken from the literature. Then, the elements of the feature maps obtained by the two paths are added together, and the weight ω of each channel is obtained by the Sigmoid function. At last, the weights ω are multiplied by the original input feature map. The calculation of the weights ω is shown in (Equation 4):


(4) $$\text{ω}=\text{σ}\left(C1D_{k}\left({\text{y}}_{1}\right)+C1D_{k}\left({\text{y}}_{2}\right)\right)$$

In (Equation 4), represents the Sigmoid activation function, C1D represents the one-dimensional convolution, K represents the one-dimensional convolution kernel size, y1 represents the feature map output by the average pooling path, y2 represents the feature map output by the standard deviation pooling path.

### 3.3 Multi-scale feature extraction module

**Figure 5:**
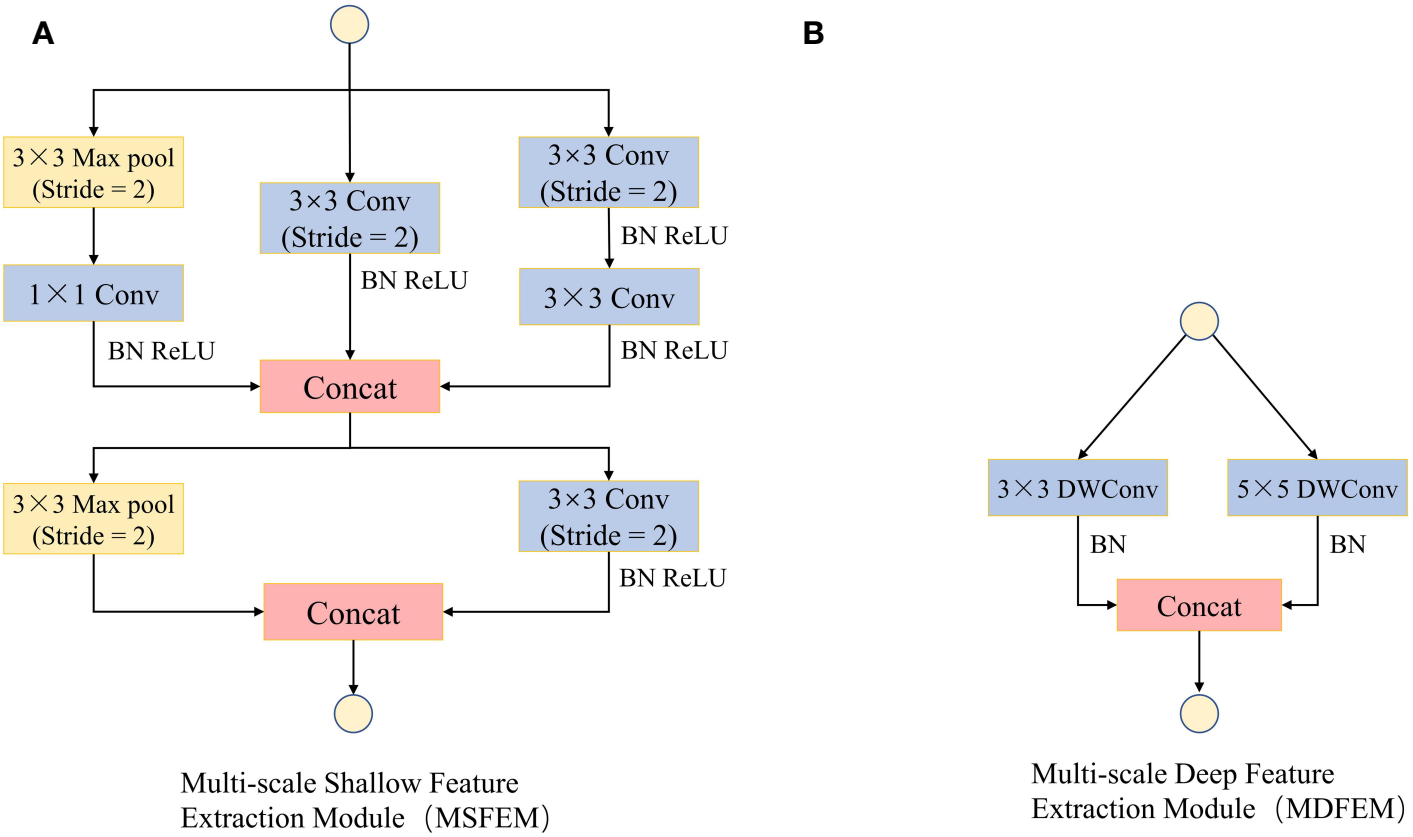
Multi-scale feature extraction module (MFEM). **(A)** Multi-scale Shallow Feature Extraction Module (MSFEM). **(B)** Multi-scale Deep Feature Extraction Module (MDFEM).

In the convolutional neural networks, the low-level convolutions mainly extract simple features such as color, texture, and edge of images, which usually have strong expressive power in local regions of images Yang et al., 2022), while the features extracted by high-level convolutions are abstract, global, and have global expressive power (Li et al., 2020). In the ShuffleNetV2 model, a 3×3 convolutional layer and a maximum pooling layer are used to extract low-level convolutional features. However, this method extracts fewer features, and the receptive field is fixed. This leads to the fact that low-level convolution cannot adequately capture the subtle feature differences of different size spots in crop leaf diseases (Shah et al., 2017). Therefore, this study designed a multi-scale shallow feature extraction module (**Figure 5A**) with a combination of a maximum pooling layer and multiple 3×3 convolutional layers to improve the response of the shallow network to features of different granularity. Meanwhile, a multi-scale deep feature extraction module (**Figure 5B**) with the combination of 3×3 convolutional layers and 5×5 convolutional layers was designed to further improve the global feature extraction capability of the model.

### 3.4 Crop leaf disease recognition model REM-ShuffleNetV2

**Figure 6:**
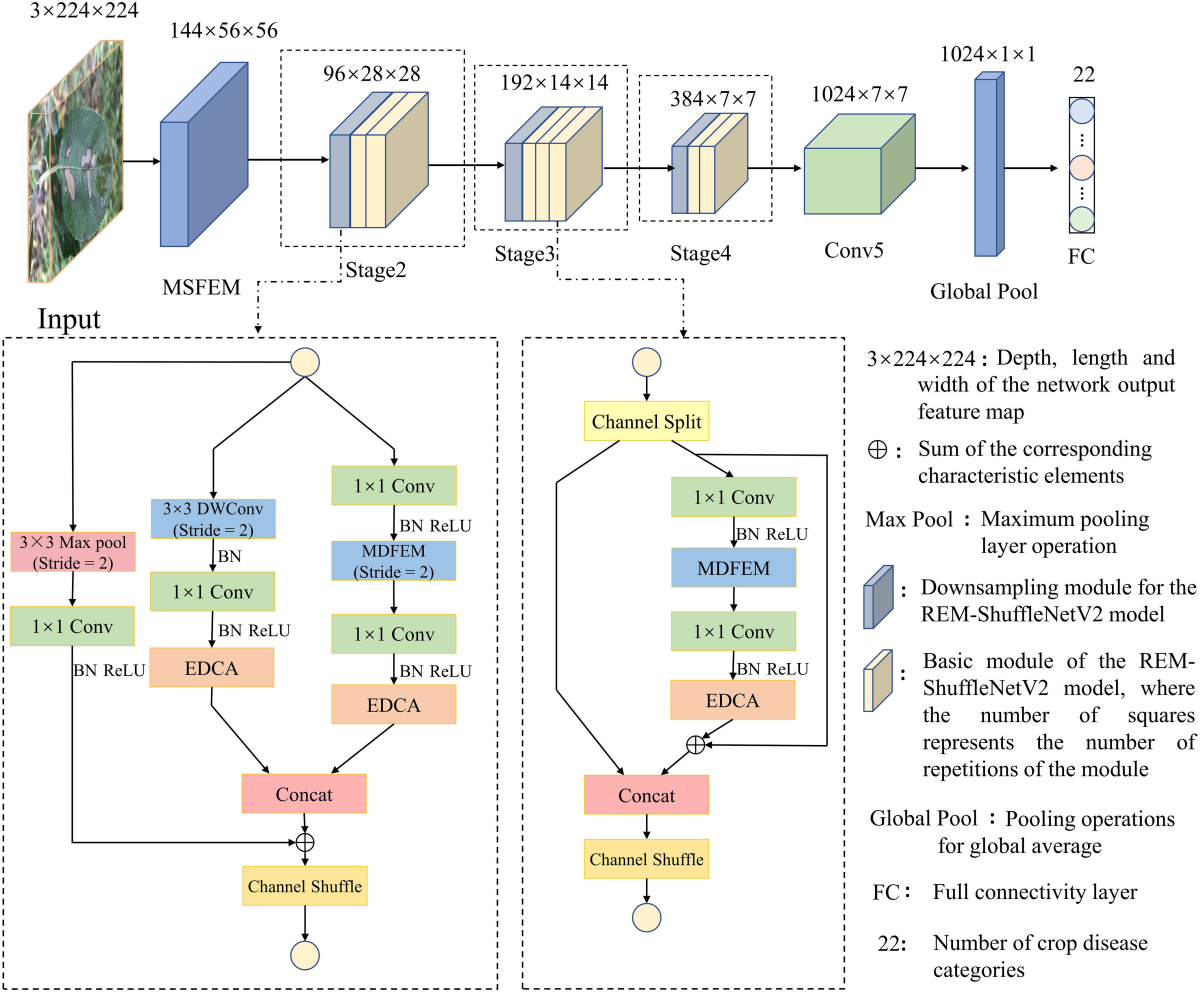
Overall model structure diagram.

ShuffleNetV2 model adopts lightweight design strategies such as depthwise convolution, channel random rearrangement, etc., which has less parameters and computation. However, the early lesions of crop leaf diseases are sparsely distributed, and the lesions tend to exhibit small area, inconspicuous features, and different morphologies, resulting in a lower overall recognition accuracy of the ShuffleNetV2 model. To further improve the accuracy of the model, this study optimized the ShuffleNetV2 model by first changing the number of repetitions of the basic modules in the Stage2, Stage3, and Stage4 phases of the model to [2, 3, 2], and fine-tuning the number of output channels to reduce the number of parameters in the model. Then, the residual structure is introduced into the basic feature extraction module of the ShuffleNetV2 model. The residual structure can increase the network learning path while preserving the original features, so that the network can pass the shallow information directly to the deep layer, solve the problem of gradient disappearance or gradient explosion that occurs in the process of model training, thereby improving the expression ability of the model (Le et al., 2023). In the residual structure of the downsampling module, the maximum pooling layer was used to complete the downsampling, and the number of channels was adjusted by 1×1 convolution, to ensure that the output number of channels was consistent. The EDCA module is introduced after the pointwise convolution at the tail of the basic feature extraction module, so that the model can pay targeted attention to the disease spot features in the input data, to improve the model’s ability to extract effective feature information. Finally, a multi-scale feature extraction module is introduced to enhance the model’s ability to extract shallow semantic information and deep semantic information. Combining the above improvement approaches, this study proposes the high-precision and low-consumption network model REM-ShuffleNetV2, as shown in **Figure 6**.

## 4 Results and analysis

### 4.1 Experimental environment setup

The experiments were conducted using a desktop computer as the processing platform, the operating system was Windows 10, and the Pytorch framework was used, the experimental environment was constructed in the Anaconda3 software, and the program was written in Python 3.8, the CUDA version was 11.1, and the Torch version was 1.8.0. Hardware: The processor is Intel Pentium G4560, the running memory is 16G, the graphics card is NVIDIA GeForce RTX3050, and the video memory is 8G.

Considering the hardware performance of the equipment and the training effect, the batch training method was used to divide the training and testing process into multiple batches, each batch contained 32 images, and the number of iterations was set to 60. The loss function uses cross-entropy loss and the classification layer uses Softmax function. The model was trained using an SGD optimizer with a momentum parameter of 0.9 and a weight decay parameter of 0.0005. The initial learning rate was 0.01, which was tuned using a cosine annealing decay strategy, with a total number of steps in a cycle of 60, and a lower value of 1e-9 for the learning rate.

### 4.2 Evaluation metrics

To evaluate the performance of the REM-ShuffleNetV2 network, this paper uses model size and number of parameters as the evaluation criteria for model complexity, and precision P, recall R, F1 score, and accuracy A on the test set as the evaluation indexes for model performance. The above four performance indicators are calculated as shown in Equations 5–8.


(5) $$\text{P}=\frac{TP}{TP\;+\;FP}$$

(6) $$\text{R}=\frac{TP}{TP\;+\;FN}$$

(7) $$\text{A}=\frac{TP\;+\;TN}{TP\;+\;TN\;+\;FP\;+\;FN}$$

(8) $${\text{F}}_{1}=\frac{2\;*\;P\;*\;R}{P\;+\;R}$$

Where TP is the result of correctly predicting positive classification; FP is the result of incorrectly prediction of positive classification; TN is the result of correctly predicting negative classification; FN is the result of incorrectly predicting negative classification.

### 4.3 ShuffleNetV2-1.0 model parameter tuning

To obtain the optimal parameters of the ShuffleNetV2-1.0 model, this study adjusted the number of basic modules and the number of output channels in the Stage2, Stage3, and Stage4 phases, designed five different parameters and conducted experiments, and the experimental results are shown in **Table 2**. Under the condition of the constant number of output channels, the best training results of the model are obtained when the number of basic modules in Stage2, Stage3, and Stage4 is [2, 3, 2], and based on this, the best model recognition results with the accuracy of 93.50% were obtained when the number of output channels of the model was [116, 232, 464, 1024]. However, with the number of output channels set to [96, 192, 384, 1024], the accuracy of the model was only 0.18% lower than the best case, but the size of the model was reduced by 22%. To consider the accuracy rate and model size, this study sets the number of basic modules in Stage2, Stage3, and Stage4 to [2, 3, 2], and the number of output channels to [96, 192, 384, 1024], and under this parameter, the accuracy rate of the model was improved by 0.48% compared with that of the original model, and the size of the model was reduced by 1.89MB. The next optimization experiments were carried out under this parameter.

**Table 2:** ShuffleNetV2-1.0 Parameter Tuning.

Number	Repeat	Output channels	Accuracy/%	Model size/MB
0	[3, 7, 3]	[116, 232, 464, 1024]	92.84	5.03
1	[3, 3, 3]	[116, 232, 464, 1024]	93.20	4.56
2	[2, 3, 2]	[116, 232, 464, 1024]	93.50	4.12
3	[1, 3, 1]	[116, 232, 464, 1024]	93.08	3.63
4	[2, 3, 2]	[96, 192, 384, 1024]	93.32	3.14
5	[2, 3, 2]	[96, 192, 384, 768]	92.96	2.74

### 4.4 Effects of different down sampling methods in residual structure on model performance

To study the effect of different down sampling methods in the residual structure of the down sampling module on the performance of the model, this study conducted comparative experiments using the completed down sampling methods of the maximally pooled layer (RM), the average pooled layer (RA), and the 3 × 3 convolutional layer (RC). The results are shown in **Table 3**, using RM and RA to complete downsampling in residual structure improves the performance of the model, this is because the pooling layer retains the main feature information of the image while completing downsampling (Saeedan et al., 2018). Among them, the best results achieved by using RM to complete the downsampling, the F1 score and accuracy of the model increased by 2.02% and 1.67% compared with the original model, this is mainly because RM, by selecting the maximum value, can select the feature activation value with the strongest response and discard the other weaker responses, realizing the downsampling of retaining the important information (He et al., 2022). The use of RC to accomplish downsampling was the least effective, with the number of parameters and model size increasing by 3.25M and 17.84MB, and the F1 score and accuracy decreasing by 0.82% and 0.48%.

**Table 3:** Experimental results for different downsampling methods in residual structure.

Model	Precision /%	Recall /%	F1 score /%	Accuracy /%	Parameters /M	Model size/MB
ShuffleNetV2	92.27	92.42	92.53	93.32	3.03	3.14
ShuffleNetV2-RM	95.00	94.31	94.55	94.99	3.39	5.17
ShuffleNetV2-RA	93.62	92.97	93.14	93.74	3.39	5.17
ShuffleNetV2-RC	92.12	91.55	91.71	92.84	6.28	20.99

### 4.5 Effects of different attention mechanisms on model performance

To verify the effectiveness of the EDCA module proposed in this study, comparative experiments are conducted with the SE module, the original ECA module, and the SRM module, respectively. **Table 4** shows that compared to the ShuffleNetV2 model, the model recognition accuracies with the introduction of the SE module, ECA module, SRM module, and EDCA module increased by 0.30%, 0.54%, 0.71%, and 0.89%, respectively; and the F1 scores increased by 0.19%, 0.17%, 0.44%, and 0.68%, suggesting that the introduction of the attention mechanism helps in the recognition of leaf diseases in crops. Meanwhile, the introduction of the EDCA module compared to the original ECA module improved the F1 score and accuracy by 0.51% and 0.35%, respectively. In addition, compared with other attention mechanism modules, the EDCA module achieves the optimal recognition effect with the number of parameters and model size basically unchanged.

**Table 4:** Experimental results of introducing different attention mechanisms into the model.

Model	F1 score/%	Accuracy/%	Parameters/M	Model size/MB
ShuffleNetV2	94.55	94.99	3.39	5.17
ShuffleNetV2+SE	94.74	95.29	3.49	5.28
ShuffleNetV2+ECA	94.72	95.53	3.39	5.18
ShuffleNetV2+SRM	94.99	95.70	3.41	5.23
ShuffleNetV2+EDCA	95.23	95.88	3.39	5.18

**Figure 7:**
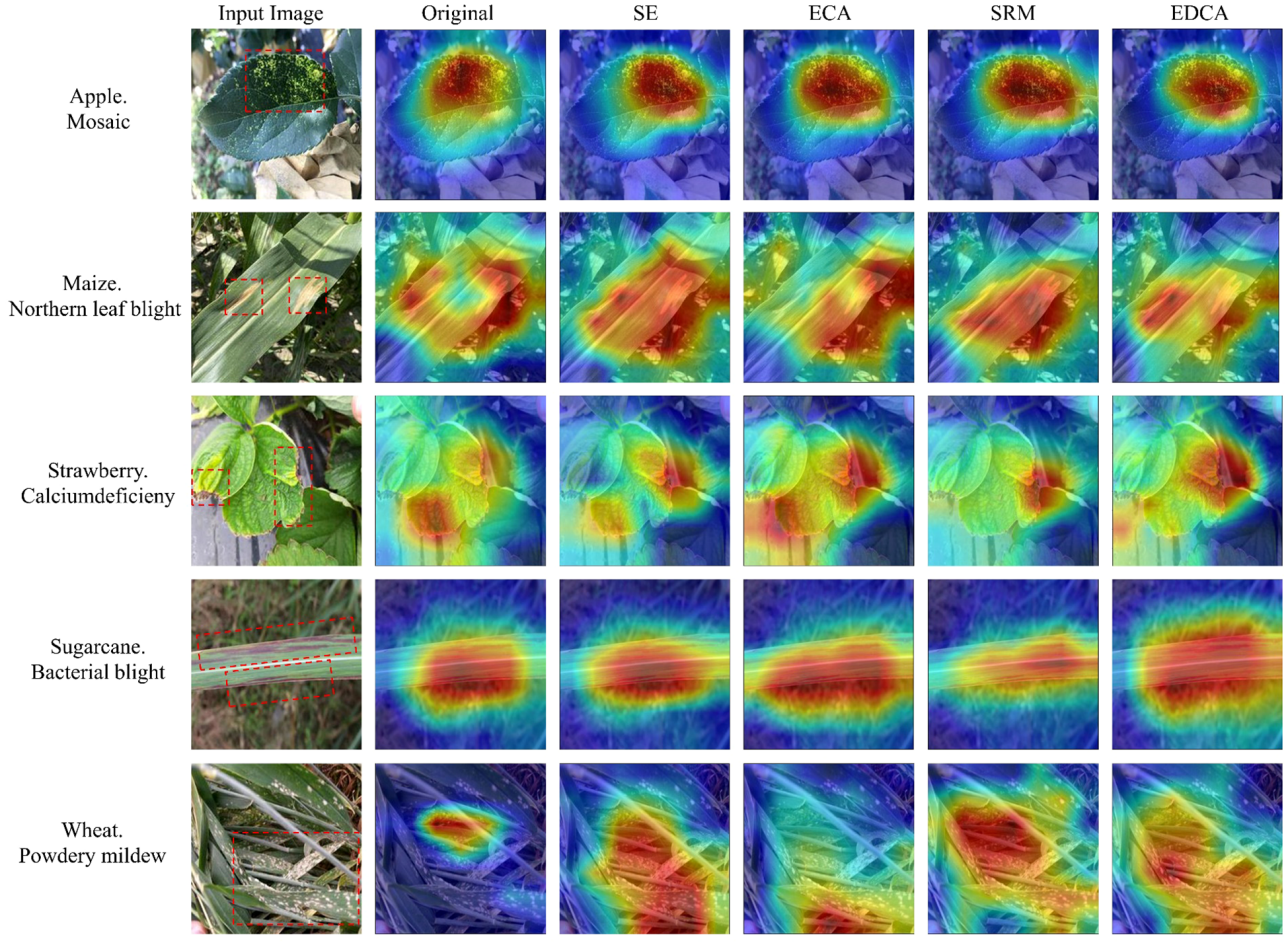
Comparison of the heatmap for different attention mechanisms. The red boxes in the original image indicate the main areas of disease in the crop.

Heatmap can intuitively show whether the network learns the key features or not through the degree of color change, this paper visualizes the feature map after the introduction of the attention mechanism in the ShuffleNetV2 model in the form of a heatmap (**Figure 7**), in which the more the color tends to be in deep red, indicating that the model is more responsive in that region. As is shown in **Figure 7**, compared with the ShuffleNetV2 model, the model incorporating the attention mechanism can better notice the feature regions related to crop disease leaves and has a stronger ability to recognize the feature information of the crop disease. Meanwhile, the introduction of the EDCA module can extract the feature information of the diseased area more accurately than other attention mechanisms, effectively avoiding the interference of non-important features such as the background environment, which further proves the effectiveness of the EDCA module.

### 4.6 Effect of MSFEM module on model performance

**Figure 8:**
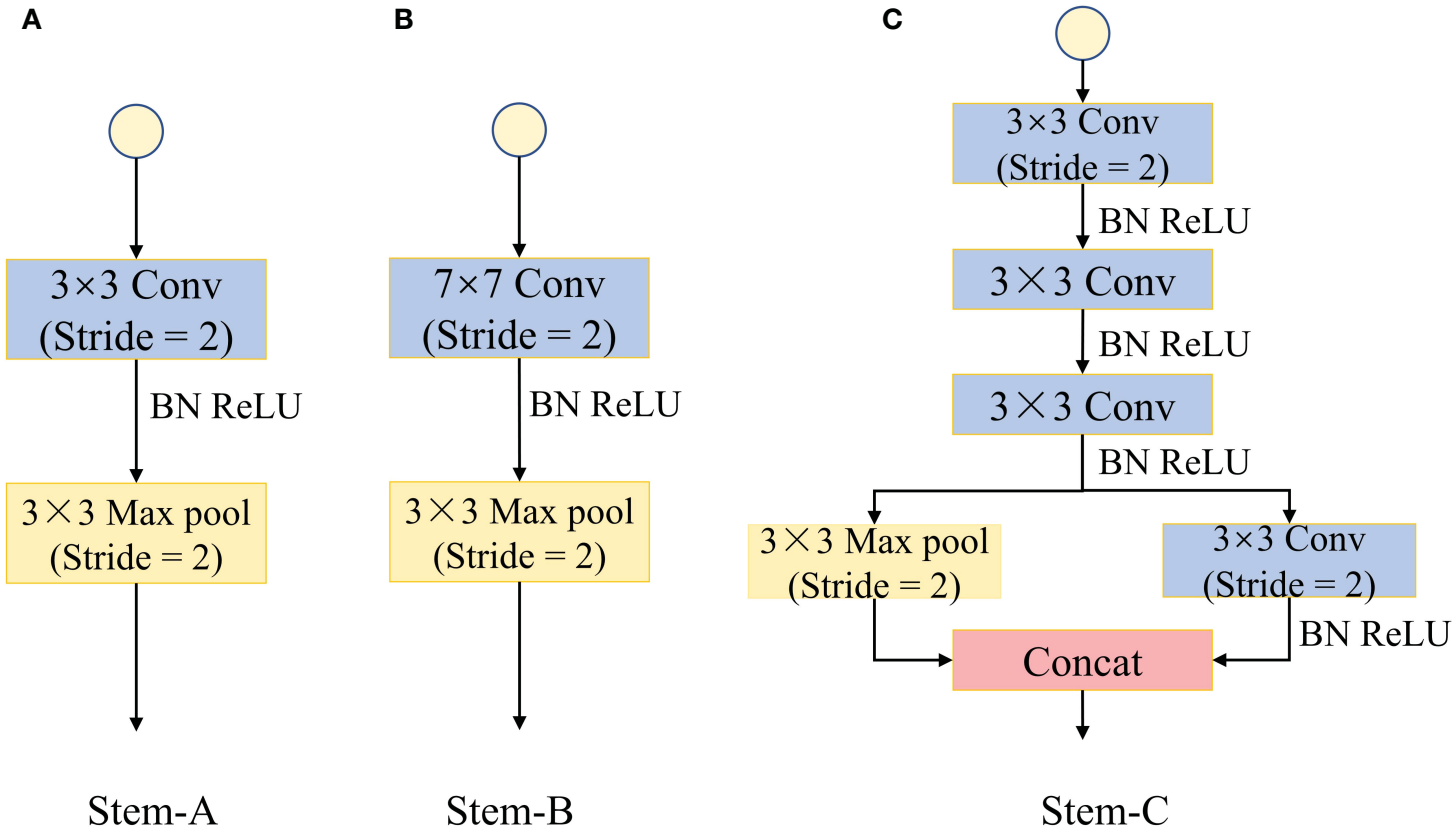
Stem module structure. **(A)** Stem-A. **(B)** Stem-B. **(C)** Stem-C.

To better extract shallow feature information, different network models are designed with different network structures. As shown in **Figure 8**, Stem-A is the shallow feature extraction module of the ShuffleNetV2 model, which consists of a 3×3 convolutional layer with a step size of 2 and a 3×3 maximum pooling with a step size of 2. Stem-B is the shallow feature extraction module of the ResNet model, which consists of a 7×7 convolutional layer with a step size of 2 and a 3×3 maximum pooling with a step size of 2. Stem-C is the shallow feature extraction module of the Inception-ResnetV2 model, which uses a stack of three 3×3 convolutional layers instead of 7×7 convolutional layers, and combines the 3×3 convolutional layers with the maximum pooling layer through a branch structure (Szegedy et al., 2017). To verify the effectiveness of the MSFEM module, a comparison experiment was conducted and the results are shown in **Table 5**. The introduced Stem-B module has basically the same number of parameters and model size compared to the original model (ShuffleNetV2-Stem-A), but the F1 score and accuracy are reduced by 0.66% and 0.38%. The introduction of the Stem-C module increased the model’s nonlinear capability and receptive fields, and the model’s F1 score and accuracy improved by 0.86% and 0.53%, respectively. The introduction of the MSFEM module increased the number of parameters and model size by 0.25M and 0.26MB, but the F1 score and accuracy improved by 1.01% and 0.77%, respectively. Taken together, the test with the introduction of the MSFEM module was the most effective.

**Table 5:** Comparison of experimental results of different shallow feature extraction modules.

Model	F1 score/%	Accuracy/%	Parameters/M	Model size/MB
ShuffleNetV2-Stem-A	92.53	93.32	3.03	3.14
ShuffleNetV2-Stem-B	91.87	92.94	3.04	3.15
ShuffleNetV2-Stem-C	93.39	93.85	3.10	3.22
ShuffleNetV2-MSFEM	93.54	94.09	3.28	3.40

### 4.7 Ablation experiment with the REM-ShuffleNetV2 model

To explore the performance enhancement of the ShuffleNetV2 model brought about by the improved approach of using architecture tuning, residual structure connection, EDCA module, and Multiscale Feature Fusion Module (MFEM), ablation experiments are conducted and the results are shown in **Table 6**. After data enhancement, the F1 score and accuracy of the ShuffleNetV2-1.0 model improved by 6.83% and 5.01%, respectively, without increased model parameters. After parameter tuning, the F1 score of the model was improved by 0.28% and the accuracy by 0.48%, while the number of parameters and model size were reduced by 1.84M and 1.89MB. When the residual structure method is introduced into the basic feature extraction module of the model, the F1 score and accuracy of the model increased by 2.02% and 1.67%, respectively, but the number of parameters and model size increased by 0.39M and 2.03MB. The introduction of the EDCA module improves the F1 score and accuracy of the model by 0.68% and 0.89% while keeping the number of parameters constant. With the introduction of the multi-scale feature fusion module, the F1 score and accuracy of the model increased by 1.39% and 0.84%, while the number of parameters and model size increased by 1.01M and 1.05MB, respectively. Finally, the F1 score and accuracy of the REM-ShuffleNetV2 lightweight model proposed in this study were 96.62% and 96.72%, which were 4.37% and 3.86% higher than the original model, the number of covariates was 4.40M which was 0.47M less than that of the original model, and the size of the model was 6.23MB which was 1.20MB more than that of the original model.

**Table 6:** Ablation experiment with the REM-ShuffleNetV2 model.

Model	Data enhancement	Parameter tuning	Residual structure	EDCA module	Multi-scale feature fusion module	F1 score/%	Accuracy /%	Parameters /M	Model size/MB
ShuffleNetV2	–	–	–	–	–	85.42	87.83	4.87	5.03
	√	**-**	–	–	–	92.25	92.84	4.87	5.03
	√	√	–	–	–	92.53	93.32	3.03	3.14
	√	√	√		–	94.55	94.99	3.39	5.17
	√	√	√	√	–	95.23	95.88	3.39	5.17
REM-ShuffleNetV2	√	√	√	√	√	96.62	96.72	4.40	6.23

“-” means not to use the improvement factor, “√” means to use the improvement factor.

**Figure 9:**
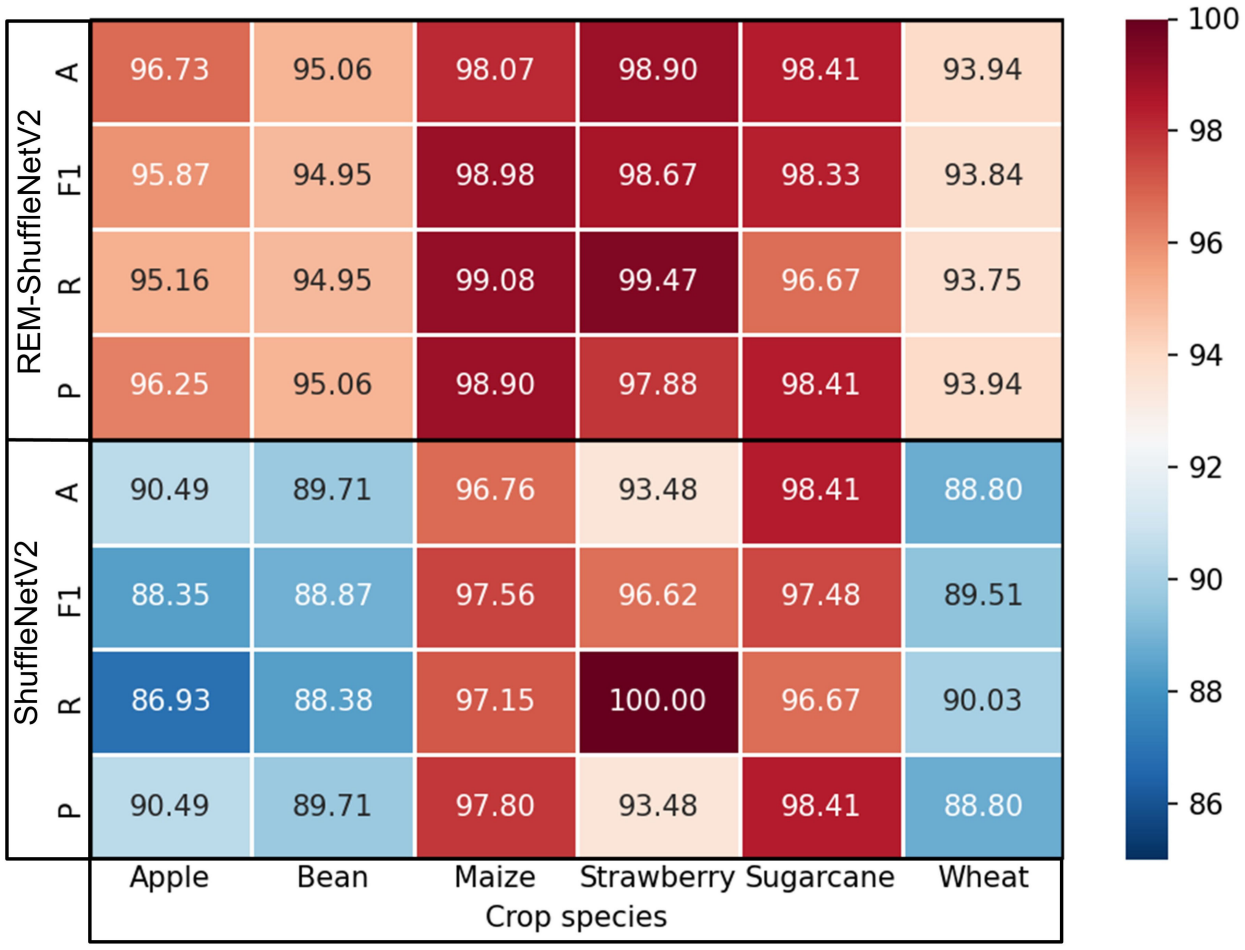
Performance metrics of the model before and after improvement on individual crops.

To observe the variation of performance metrics of ShuffleNetV2 model and REM-ShuffleNetV2 model on different crop diseases, the precision P, recall R, F1 score, and accuracy A of the models were visualized for different crops. As shown in **Figure 9**, the recognition effect of the ShuffleNetV2 model on apple disease, soybean disease, and wheat disease was poor, this is because they have more types of diseases and high similarity of lesion characteristics, which leads to recognition difficulties. The recognition effect of ShuffleNetV2 model on maize disease, strawberry disease, and sugarcane disease was better, this is because they are easy to differentiate due to their fewer types of diseases and distinct disease characteristics. REM-ShuffleNetV2 improved crop disease recognition to varying degrees. On the more difficult to recognize apple, bean, and wheat diseases, the average F1 score and average accuracy improved by 7.52% and 6.24%, 6.08% and 5.35%, 4.33% and 5.14%, respectively, compared to the original model. For the easily recognized maize disease and strawberry disease, the average F1 score and average accuracy improved by 1.42% and 1.31%, 2.05% and 5.42%, respectively, compared with the original model. For sugarcane diseases, the average precision, average recall, and average accuracy of the REM-ShuffleNetV2 model were the same as those of the original model, but the average F1 score was improved by 0.85%.

### 4.8 Different network comparison experiments

**Figure 10:**
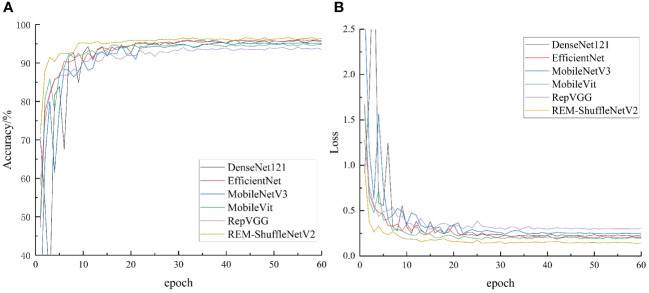
Accuracy and loss of comparative network model. **(A)** Accuracy; **(B)** Loss value.

To further verify the effectiveness of the REM-ShuffleNetV2 model, this paper compared it with the DenseNet121 (Huang et al., 2017), EfficientNet (Tan and Le, 2019), MobileNetV3 (Howard et al., 2019), MobileVit (Mehta and Rastegari, 2021) and RepVGG (Ding et al., 2021) models under the same test conditions. The change curves of accuracy and loss value of different models are shown in **Figure 10**.

As can be seen in **Figure 10A**, after 60 iterations, the accuracy of each model in crop disease tends to stabilize, which indicates that the performance of the model has been fully demonstrated. REM-ShuffleNetV2 is the fastest converging model among these models. When iterating to the 5th round, the accuracy of the REM-ShuffleNetV2 model had already reached 90%. As the iteration proceeds, the accuracy of the model reaches 96% at round 20 and begins to converge. In contrast, the training curves of the remaining models behave similarly. After 15 rounds of iterations, these models all achieve 90% accuracy and begin to converge after 30 rounds. In the later stages of training, the REM-ShuffleNetV2 model exhibits higher accuracy with less fluctuation. This shows that REM-ShuffleNetV2 had stronger robustness and faster convergence on the crop leaf disease test set. **Figure 10B** shows that the loss value of REM-ShuffleNetV2 decreases the fastest and obviously, and at 20 rounds of iteration, the loss value basically stabilizes, and the network loss value maintains around 0.146. From the perspective of loss-value convergence, the REM-ShuffleNetV2 model is ideally trained. Other measures of the model are shown in **Table 7**.

**Table 7:** Performance comparison results of different models.

Model	Precision/%	Recall/%	F1 score /%	Accuracy/%	Parameters/M	Model size/MB
DenseNet121	95.69	94.99	95.30	96.00	26.61	27.21
EfficientNet	95.67	94.82	95.17	96.06	15.40	15.69
MobileNetV3	95.01	94.49	94.69	95.29	16.14	16.34
MobileVit	93.84	93.66	93.61	95.05	7.41	7.56
RepVGG	94.11	93.03	93.41	94.09	29.97	30.18
REM-ShuffleNetV2	96.85	96.48	96.62	96.72	4.40	6.23

As shown in **Table 7**, compared to the conventional models DenseNet121 and RepVGG, the REM-ShuffleNetV2 lightweight model had higher accuracy and F1 scores, and the number of parameters was significantly reduced. Compared with the lightweight convolutional networks EfficientNet, MobileNetV3, and MobileVit, the number of parameters of the REM-ShuffleNetV2 model were only 28.57%, 27.28% and 59.38% of those of EfficientNet, MobileNetV3 and MobileVit, but the model’s F1 scores and accuracy are 1.45% and 0.66%, 1.93% and 1.43%, 3.01% and 1.67% higher than them respectively. In summary, the REM-ShuffleNetV2 model achieves good performance in terms of performance and complexity.

### 4.9 Analysis of model robustness performance

**Figure 11:**
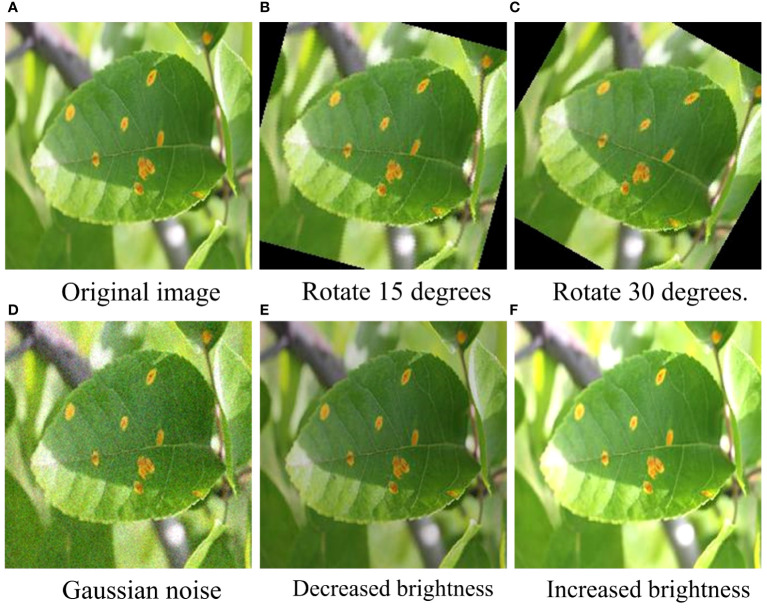
Example plots under different treatments. **(A)** Original image. **(B)** Rotate 15 degrees. **(C)** Rotate 30 degrees. **(D)** Gaussian noise. **(E)** Decreased brightness. **(F)** Increased brightness.

To further verify the anti-interference ability of the REM-ShuffleNetV2 model, we performed a variety of treatments on the test set, including adding Gaussian noise, performing a rotation process, and adjusting the luminance to simulate more realistic environmental conditions (as shown in **Figure 11**). These treatments help to evaluate the performance of the models in the face of complex, variable environments and thus provide a more complete picture of their robustness and reliability. The classification accuracy of each model under different treatments is shown in **Table 8**.

**Table 8:** Classification accuracy of each model under different treatments.

Model	A	B	C	D	E	F
DenseNet121	96.00	90.69	85.20	94.63	90.45	92.36
EfficientNet	96.06	90.45	83.53	93.32	90.69	94.27
MobileNetV3	95.29	80.85	72.85	88.42	88.07	93.50
MobileVit	95.05	88.60	82.70	89.68	91.83	93.20
RepVGG	94.09	72.85	59.55	88.96	88.60	88.31
REM-ShuffleNetV2	96.72	91.53	86.87	89.68	91.64	94.87

A, B, C, D, E, and F in the table correspond to the different treatments of a, b, c, d, e, and f in **Figure 11**, respectively. The data in the table are in %.

**Table 8** shows that the classification accuracy of each model generally decreases more significantly when Gaussian noise and 30-degree rotation treatments are added. Under Gaussian noise processing, the recognition effect of the REM-ShuffleNetV2 model is significantly worse than that of the DenseNet121 model and the EfficientNet model; while under the brightness reduction processing, the recognition effect of the REM-ShuffleNetV2 model is slightly lower than that of the MobileVit model. However, under other conditions of processing, the recognition effect of the REM-ShuffleNetV2 model is better than the other models. Taken together, REM-ShuffleNetV2 still shows good recognition results under different treatments, showing good robustness.

### 4.10 Confusion matrix for different models

**Figure 12:**
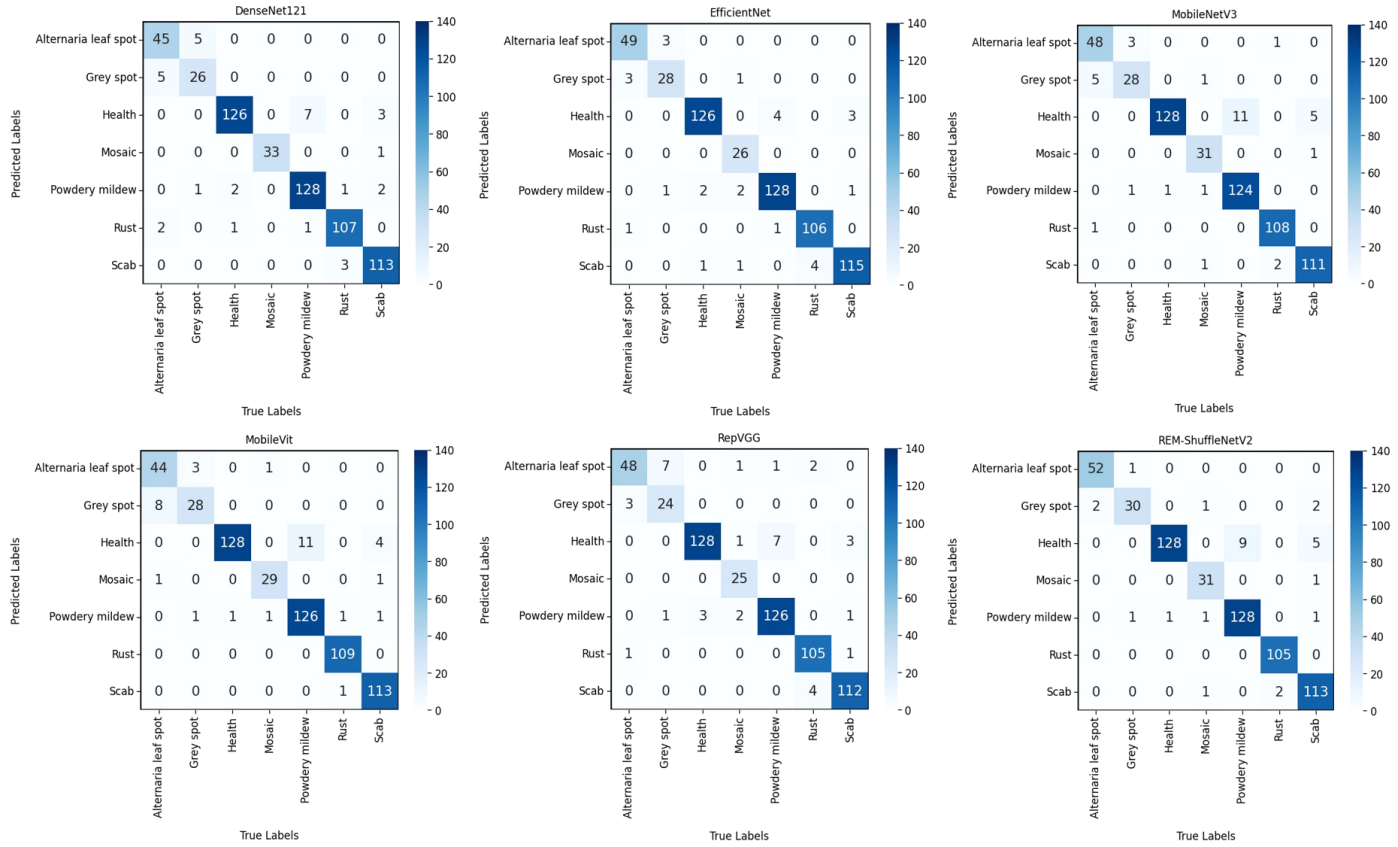
Confusion matrix of different models.

The confusion matrix is usually used as an evaluation metric for machine learning classification models, which can demonstrate the number of observations that are misclassified and right-classified by the model, thus assessing the performance of the model (Bi et al., 2023). In the dataset used in this experiment, the types of apple leaf diseases are the most numerous, and different apple leaf diseases only have slight differences in a certain localization, which is characterized by “high within-class variance and low between-class variance”, therefore, the confusion matrix of apple leaf diseases was used to present the results, as shown in **Figure 12**.

From **Figure 12**, Alternaria leaf spot and gray spot are easily confused because of their high spot similarity, while scab and powdery mildew are easily confused with healthy leaf because their early spot characteristics are not obvious and basically indistinguishable from those of healthy leaf, which leads to misclassification in the model. The REM-ShuffleNetV2 model performed well in the identification of confusing apple leaf diseases with a number of recognition errors of 28, which was comparable to the Efficientnet model. Compared with the DenseNet121, MobileNetV3, MobileVit, and RepVGG models, the REM-ShuffleNetV2 reduced 6, 5, 7, and 10 recognition errors, respectively.

## 5 Discussion

Advanced convolutional neural networks are often designed to be deep and wide to learn patterns of features from different objects. However, in the crop leaf disease images used in this paper, the disease features are similar and scattered, and no obvious patterns exist to be learned. Therefore, blindly stacking the number of network layers and increasing the model width may overfit useless feature information without improving the performance of the model. On the contrary, doing so may increase the number of parameters and computational effort of the model, thus affecting the efficiency and usefulness of the model (Peng and Li, 2023). In this study, it was found that properly reducing the parameters of the model did not degrade the model performance, but rather improved it. This indicates that appropriately reducing the number of parameters of the model helps the model learn features better. Therefore, reducing the number of parameters of the model appropriately for a specific task and dataset may be an effective strategy to help improve the performance and generalization of the model.

In the task of image classification, the region of interest is often distributed in multiple regions of the image, and more global information and higher-level feature information are needed to better recognize the target. The smaller the receptive field is, the smaller the range of the original image to which it corresponds, which means that it contains features that tend to be more localized and detailed, and the high-level semantic information used to deal with the complex task is difficult to be captured by the network; the larger the receptive field is, the larger the range of the original image to which it corresponds, which means that it contains more global and higher semantic level features. In the real environment, crop diseases have problems such as different sizes of spots and a wide range of disease distribution. In this paper, a multi-scale feature extraction module is introduced to enhance the model’s ability to extract feature information at different scales and to solve the problem of losing small feature information due to downsampling. To further improve the model performance, this paper draws on the idea of ResNet and introduces a residual structure to overcome the problems of gradient vanishing and gradient explosion during network training, to better fit the data.

Attentional mechanisms are often used to improve the performance of models by better aggregating information about the features of the network model for the region of interest and reducing the influence of extraneous background (Sun et al., 2022; Liao et al., 2023). However, different attention mechanisms work differently and have different impacts on model performance. Compared with other attention mechanisms, the introduction of the EDCA module designed in this paper can effectively improve the performance of the ShuffleNetV2 model for crop leaf disease recognition. This is because the attention module uses two different pooling layers to couple the global information and a local cross-channel interaction strategy without dimensionality reduction to obtain more accurate attention information by aggregating the cross-channel information with a one-dimensional convolutional layer.

Although the study has achieved some results, there are still some limitations. Firstly, the sample images used in the experiment were taken under real environments on sunny or cloudy days, and although realistic factors were taken into account to a certain extent, further in-depth research is needed to fully reflect the performance under various environmental conditions. Secondly, due to the limitation of shooting conditions, the types of disease samples collected are limited, which limits the application range of the model to a certain extent. In future work, we will collect more plant disease data from real scenarios, covering different types, parts and developmental stages of the disease, and develop more efficient and accurate deep learning models to be able to differentiate between more types of crop disease. In addition, we try to deploy the model to cell phones to help farmers find diseases on plants in time so that they can take appropriate control measures to prevent the spread of diseases. In addition, we also plan to deploy it into field management robots for real-time monitoring of crop diseases. This will help professionals understand the type, distribution and severity of diseases and develop more effective disease management strategies.

## 6 Conclusions

Aiming at the problems of low recognition accuracy and complex model structure of existing models, this paper proposes a lightweight crop leaf disease recognition model REM-ShuffleNetV2. First, we build a field crop disease dataset, which contains 22 categories of 6 crops with a total of 8408 sample images. To reduce the complexity of the model, architectural adjustments were made to the ShuffleNetV2 model. The residual structure was introduced in the basic feature extraction module, which solved the problem of the model’s gradient disappearing during the training process and improved the convergence speed of the model. To improve the model’s ability to extract effective features in complex backgrounds, we used the EDCA module to filter out the complex interference information in the samples. Meanwhile, we also introduced the MSFEM module and MDFEM module designed in this paper to improve the model’s ability to extract feature information at different scales. Finally, the REM-ShuffleNetV2 model achieved 96.72% recognition accuracy on the crop leaf disease test set, which increased by 3.86% compared to the ShuffleNetV2 model.

In order to further evaluate the performance of the REM-ShuffleNetV2 model, we conducted comparison experiments with the DenseNet121, ResNet18, MobileNetV2, and GhostNet models. The experimental results show that the recognition accuracies of the REM-ShuffleNetV2 model were 0.72%, 1.67%, 2.09%, and 11.52% higher than these models, while the model structure was more streamlined. In addition, the superiority of the REM-ShuffleNetV2 model in fine-grained classification is further demonstrated by the analysis of the confusion matrix.

## Data availability statement

The dataset and code in this study can be accessed by contacting the corresponding author.

## Author contributions

HZ: Conceptualization, Methodology, Writing – original draft, Writing – review & editing, Funding acquisition. JC: Methodology, Data curation, Formal analysis, Writing – original draft. XN: Project administration, Writing – review & editing. ZD: Supervision, Visualization, Writing – review & editing. LQ: Software, Investigation, Writing – review & editing. LM: Supervision, Writing – original draft. JL: Software, Validation, Writing – original draft. YS: Investigation, Data curation, Writing – original draft. QW: Writing – review & editing, Project administration, Supervision.
